# Air ambulance flights in northern Norway 2002-2008. Increased number of secondary fixed wing (FW) operations and more use of rotor wing (RW) transports

**DOI:** 10.1186/1865-1380-4-55

**Published:** 2011-08-30

**Authors:** Jan Norum, Trond M Elsbak

**Affiliations:** 1 Department of Clinical Medicine, Faculty of Health Sciences, University of Tromsø, N-9037 Tromsø, Norway; 2 Department of Oncology, University Hospital of North Norway, N-9038 Tromsø, Norway; 3 Northern Norway Regional Health Authority trust, N-8038 Bodø, Norway; 4 Helgeland Hospital Trust, Sandnessjøen, P.O.B. 613, 8801 Sandnessjøen, Norway

## Abstract

**Background:**

Air ambulance service in Norway has been upgraded during the last years. European regulations concerning pilots' working time and new treatment guidelines/strategies have called for more resources.

**Aims:**

The objective was to describe and analyse the two supplementary air ambulance [fixed wing (FW) and rotor wing (RW)] alternatives' activity during the study period (2002-2008). Furthermore we aimed to compare our findings with reports from other north European regions.

**Methods:**

A retrospective analysis. The air ambulance fleet's activity according to the electronic patient record database of "Luftambulansetjenesten ANS" (LABAS) was analysed. The subject was the fleet's operations in northern Norway, logistics, and patients handled. Type of flight, distances, frequency, and patients served were the main outcome measures.

**Results:**

A significant increase (45%) in the use of RW and a shift in FW operations (less primary and more secondary) were revealed. The shift in FW operations reflected the centralisation of several health care services [i.e. percutaneous cardiac intervention (PCI), trauma, and cancer surgery] during the study period. Cardiovascular disease (CVD) and injuries were the main diagnoses and constituted half of all operations. CVD was the most common cause of FW operations and injuries of the RW ones. The number of air ambulance operations was 16 per 1,000 inhabitants. This was more frequent than in other north European regions.

**Conclusions:**

The use of air ambulances and especially RW was significantly increased during the study period. The change in secondary FW operations reflected centralisation of medical care. When health care services are centralised, air ambulance services must be adjusted to the new settings.

## Introduction

Northern Norway covers half of Norway's land area and constitutes in size about two-thirds of that of the UK. Including the sea (Norwegian and Barents Sea) areas under Norwegian surveillance, the figures are significantly increased [1]. The people of northern Norway are scattered and the total population is only about 460,000 inhabitants. One fourth of the population are citizens of Bodø and Tromsø. About 2,500 people live in the Norwegian Arctic (Svalbard, Bear Island, Jan Mayen, and Hopen). The main industry in the region is fishery, and many fishermen are on board vessels in the Norwegian and Barents Sea. Oil and gas production is a growing business in northern Norway. The significant distances between the populated areas have been a constant challenge to the specialised health care service in terms of costs and logistics. In 2010, the Northern Norway Regional Health Authority (NNRHA) trust will spend Norwegian krone (NKr) 337 million (Euros €43 million) on air ambulance operations. The amount accounts for 3% of the total budget.

Northern Norway has a subarctic and arctic climate that introduces several challenges, especially during winter time. Cold and rough weather conditions, long distances, seasonable darkness, and snow have to be handled.

During the last decade several factors have introduced an increased pressure on the air ambulance resources. The European Union (EU) has introduced new restrictions on pilots' duty. Furthermore, new treatment methods and guidelines have been introduced. The implementation of percutaneous coronary interventions (PCI) in the treatment of cardiovascular disease at the University Hospital of North Norway (UNN) trust in Tromsø is such an example. The regional trauma centre established at the same place is another one. Similarly the care of severely injured patients has been mainly centralised to the hospitals in Bodø and Tromsø, respectively. A new guideline for the care of ischemic stroke has called for emergency radiological diagnostics followed by immediate thrombolytic care. These changes have caused an increased pressure on the air ambulance resources. With this background, we aimed to analyse and describe the changes taken place during the last decade and compare our findings to those of other north European regions.

## Materials and methods

**Figure 1:**
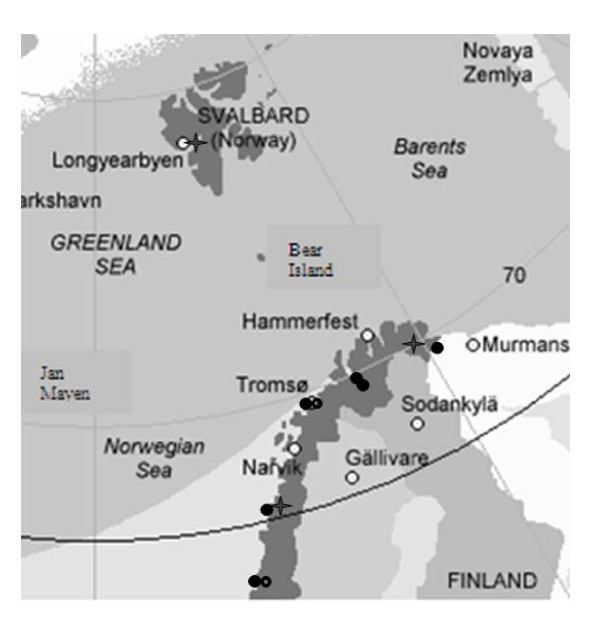
**The figure shows the locations of the air ambulance services in northern Norway**. Figure from reference [14]. Stars = search and rescue helicopter. Filled circles = airplane ambulance. Open circles = helicopter ambulance.

To meet people's expectations, the NNRHA trust has 11 air ambulance resources [6 planes (Beechcraft King Air 2002/B200), 2 ambulance helicopters (AH) (Augusta AW 139), and 3 search and rescue helicopters (SRH) (2 Sea Kings and 1 Super Puma)] scattered (7 locations) within the region. Details are shown in Figure 1. The SRHs are run by the Norwegian Air Force and the others administered by Luftambulansetjenesten ANS (http://www.luftambulanse.no). The fixed wing (FW) resources are manned with a pilot, co-pilot, and registered nurse with basic education in anesthesia and/or intensive care and special education in flight medicine. The rotor wings (RW) are manned as follows: The AHs are midsized helicopters with a crew consisting of a pilot, rescuer, and anaesthesiologist. The SRHs are manned with a crew of six members (pilot, co-pilot, engineer, navigator, rescuer, and anaesthesiologist). The ambulance helicopters are equipped with advanced medical equipment permitting a high degree of emergency and intensive care. The helicopters are mostly used outside urban areas because ground ambulances are often quicker to the incident site in urban areas. In the northern part of Norway, the flying distances may be so long that refueling may be necessary for a helicopter. This makes transport with aircraft, or "fixed wing", an attractive alternative for inter-hospital transports. The advantage is higher speed (2-2.5 times faster than a helicopter), "all weather" capacity, and no refueling. The disadvantage is the necessary secondary transport with ground ambulances between hospitals and airports. Thus RW and FW resources are supplementary to each other and not competing alternatives.

The main objective of this study was to describe and analyse the two supplementary air ambulance [fixed wing (FW) and rotor wing (RW)] alternatives' activity during the study period (2002-2008). Furthermore we aimed to compare our findings with reports from other north European regions. The air ambulance fleet's activity is prospectively registered in the electronic patient record (EPR) system of the Luftambulansetjenesten ANS (LABAS) (http://www.luftambulanse.no) by the medical crew (nurse or medical doctor). In February 2010, the LABAS database was analysed retrospectively focusing on the time period from 1 January 2002 until 31 December 2008. The data were collected by Trond M. Elsbak in cooperation with the staff at the Luftambulansetjenesten ANS. The following data were collected using a specific report sheet.

- Flight data: Date and time of start and end of task, time spent, state of emergency (non-urgent, urgent, emergent) according to the Norwegian Index for Medical Emergency Assistance, and destination (hospital) [2].

- Patient data: Sex, age, nationality, diagnosis (according to the international classification of diseases, ICD), oxygen support, intubation, analgesics given, state of seriousness [National Advisory Committee on Aeronautics (NACA) scale], intravenous administration, and the use of vasopressor drugs.

Boat and car ambulance operations were excluded from the study. The latter has been dealt with in a prior publication [3].

Our hypothesis was that the continuous centralisation of advanced specialised health care (i.e. PCI, cancer surgery, and trauma care) to the major hospitals has increased the need for and use of air ambulance resources. An equal impact on FW's and RW's activity was anticipated. In this study, we also aimed to obtain more knowledge of the service from an administrative point of view and create a foundation for future research. Furthermore, we aimed to describe the service performed in terms of patient and flight logistics together with trends.

### Statistical analysis and authorisation

The Microsoft Office Excel 2007, Microsoft Corp., Redmond, WA, was employed for the calculations and database. Statistical Package for Social Science (SPSS) version 16.0, SPSS Inc., Chicago, IL, was employed for statistical analyses. Cases with an unknown value for a particular variable were excluded from analysis involving that variable. Statistical analyses were performed employing descriptive statistics and one-way analysis of variance (ANOVA).

We accessed anonymous annual data from each air ambulance location. We had no access to individual patient identifiable data. Approval from the Regional Committees for Medical and Health Research Ethics (REK) was therefore not requested.

When mentioned, cost was reported in Norwegian krone (NKr) and converted into Euros (€) at a rate of 1€ = 7.8350 NKr as of the 11 June 2010 (http://www.norges-bank.no).

## Results

The inhabitants of northern Norway had a high consumption of FW and RW resources (16 operations/1,000 inhabitants/year) and the consumption increased (from 15 to 18 operations/1,000 inhabitants/years) during the study period. Whereas the number of RW operations increased significantly (45% AHs, 54% SRHs), there was a minor (9%) change in the use of FWs. The annual figures are shown in Table 1. The total figures in 2008 reached 7,745 operations [airplane 6,007, ambulance helicopter (AH) 1,243, search and rescue helicopter (SRH) 495]. The corresponding total flight time was 8,045 h. Thus the air ambulances are airborne on average 1 h 2 min per task. The air ambulance operations performed by the two Sea Kings (SRHs) accounted for one third of the total RW activity, and the busiest months were July and August. The number of FW operations with two patients on board rose by 14% and reached 38% at the end of the study period. The increased number of inter-hospital transports (secondary operations) improved the coordination of transfers.

**Table 1:** The table shows the annual number of fixed wing (FW) and rotor wing (RW) missions during the study period (2002-2008)

	Year						
Air ambulance	2002	2003	2004	2005	2006	2007	2008
*Rotor wing (RW) total*	*1,419*	*1,498*	*1,654*	*1,797*	*1,845*	*1,904*	*2,129*
Brønnøysund*	396	423	433	551	543	473	470
Tromsø*	460	510	626	669	671	670	773
Banak & Bodø**	563	565	595	577	631	761	886
*Fixed wing (FW) total*	*5,535*	*5,773*	*5,916*	*5,959*	*5,784*	*5,829*	*6,007*
Kirkenes	993	1,018	962	991	1,004	1,020	1,002
Alta (2 FWs)	1,585	1,460	1,570	1,717	1,649	1,752	1,770
Tromsø	863	1,080	1,210	1,189	1,092	1,078	1,123
Bodø	1,128	1,236	1,293	1,298	1,252	1,243	1,259
Brønnøysund	966	979	881	764	787	736	853
*Total (RW and FW)*							

*Ambulance helicopter (AH), **search and rescue helicopter (SRH).

Despite a significant number of transports, no accident occurred, and none of the crew members were lost during the study period.

The RWs transported patients were somewhat younger (mean age SRHs 48 years, AHs 47 years) than those handled by FWs (mean age 55 years), but the difference was not statistically significant.

Whereas the delay in RW (AH) operations varied between locations (68-84%), the RW-SRH crew did not report data. This was probably due to Norwegian Air Force regulations. However, the technical regularity was 99%. Concerning FW operations, the share of each urgency (emergent, urgent and not urgent) group was stable during the study period.

**Figure 2:**
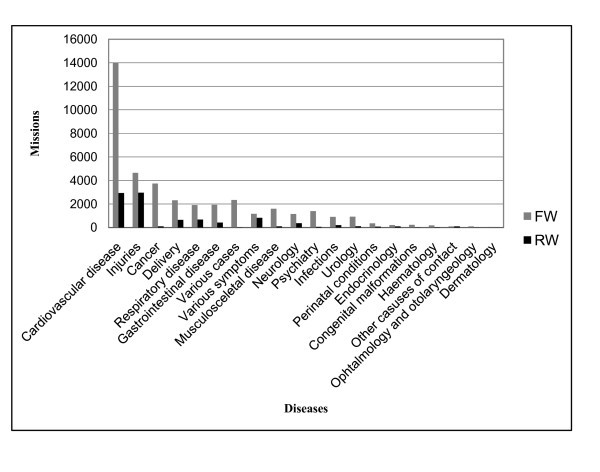
**The figure shows the number of operations with regard to diagnosis**. This is according to rotor wing (RW) or fixed wing (FW) transportation.

**Figure 3:**
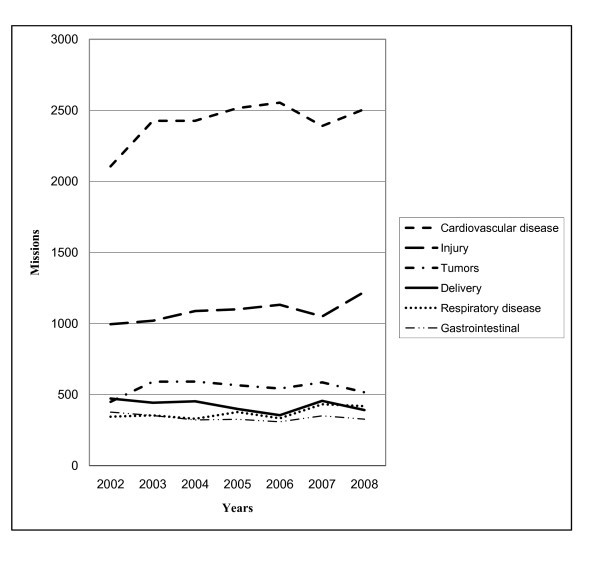
**The figure visualizes the number of flights [rotor wing (RW) and fixed wing (FW)]**. Data were taken during the study period (2002-2008) associated with the six most common diagnoses. RW = Rotor wing (helicopter). FW = Flight wing (airplane)

The most common diagnoses were coronary heart disease, injuries, cancer, delivery, respiratory disease, and gastrointestinal disease. An overview of the diagnoses with regard to RW or FW missions and number of transports are shown in Figure 2. Whereas coronary heart disease was the most common diagnosis in FW operations, injuries was the most frequent one in the RW setting. Cardiovascular disease (CVD) and injuries were the main culprits causing raised activity and constituted together half of all patients. During the study period, the number of missions due to CVD, injuries, respiratory disease, and cancer rose by 19%, 23%, 21%, and 15%, respectively. The number of transports related to delivery and gastrointestinal disease were reduced by 17% and 13%, respectively. The trends are shown in Figure 3.

Whereas most of the RW operations are urgent or emergent (92-95% of cases), FW activities are usually (61%) non-urgent. Details are shown in Table 2. This is because FW operations often are planned inter-hospital transports. Even inter-hospital transports of patients suffering from acute myocardial infarction (MI) may be non-urgent. Patients suffering from ST-elevated myocardial infarction (STEMI) are frequently hospitalised at the local hospital and treated with thrombolytic therapy. When ECG changes normalise due to therapy, patients may be transported to the university hospital for PCI in a non-urgent setting.

**Table 2:** The table shows the various stages of emergency according to operations performed by the fixed wings (FW) and rotor wings (RW), respectively

Variable		FW	RW (AHs)	RW (SRHs)
Emergency status	Non-urgent	61%	5%	8%
	Urgent	21%	45%	35%
	Emergent	18%	50%	57%

FW = Fixed wing = airplane. RW = Rotor wing [rescue and search helicopters (SRHs) and ambulance helicopters (AHs)].

**Figure 4:**
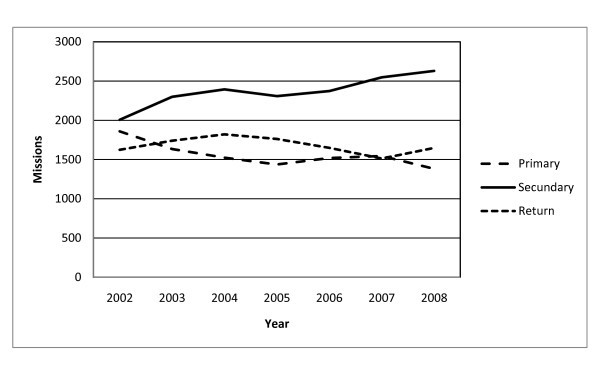
**The figure illustrates the trend in fixed wing activities**. This was according to primary, secondary and return missions during the study period (2002-2008). Primary operations = transporting patients from patient's place of living to hospital; secondary operations = inter-hospital transports; return = transport of patient back home.

A shift in air ambulance transport was revealed during the study period. Whereas 34% of all FW operations were "primary operations" (patients transported from place of living to hospital) in 2002, the figure was 24% in 2008. The "secondary operations" (inter hospital transports) increased from 36% to 46%. The shift is illustrated in Figure 4.

Looking at the National Advisory Committee on Aeronautics (NACA) score, there were minor differences between the three types of operations. The mean scores (0-7 scale) were: 3.2, primary operations; 3.6, secondary operations; 3.0, returns; respectively.

**Figure 5:**
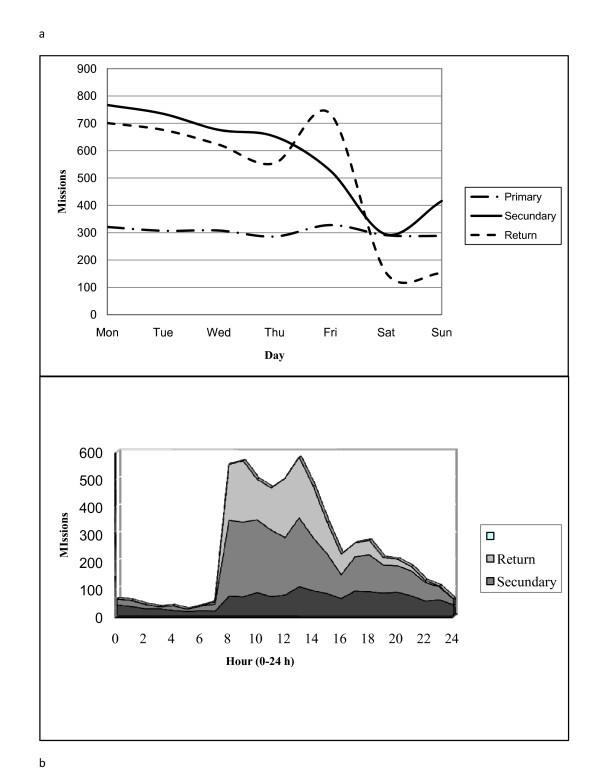
**The figure illustrates the fixed wing (airplane) activity during the week (a) and during 24 h (b)**. Most activities took place during the daytime, Monday to Friday.

Looking for strategies to improve cost-effectiveness, we revealed available resources during night and weekends. Whereas the FWs are manned day and night all week, the activity varied significantly during 24 h. Night flights are generally associated with increased risk, especially during winter time with seasonable darkness. However, during summer time the midnight sun offers "daylight facilities". Whereas the numbers of primary transports were stable through the week, there was a significant drop in secondary operations and returns during weekends. Details are illustrated in Figure 5a and 5b. Furthermore, we identified an underutilised location in the southern region located FW in Brønnøysund in which approximately half of the flights left or returned to Brønnøysund without any patients onboard. A simulation model (unpublished analysis from the Luftambulansetjenesten ANS, Bodø) indicated, due to better patient logistics, a saving of 11 million NKr (€1.4 million) by simply moving this plane to the base in Bodø.

## Discussion

Significant resources were employed in the air ambulance services in our region. According to the literature the gains related to RW operations have been inconsistent. The first major comparison of outcome in the air transport of trauma patients was published by Baxt and Moody in 1983 [4]. The study was a retrospective TRISS-based analysis with a cohort of patients transported by ground as a control group. The authors showed a 52% reduction in mortality with aeromedical treatment (AMT). Mitchell and coworkers at the Dalhousie University in Halifax, Nova Scotia evaluated the transport of trauma patients with an injury severity score (ISS) ≥12 by a dedicated RW air medical service or a standard ground ambulance [5]. They concluded that RW was associated with significantly better outcomes than the control group. Ringburg and colleagues [6] reviewed the literature and concluded helicopter emergency medical services (HEMS) saved 2.7 additional lives per 100 HEMS deployments. The positive effect of HEMS has also been confirmed by McVey et al. comparing air versus ground transport of major trauma patients [7]. Snooks and coworkers analysed the Helicopter Emergency Ambulance Services (HEAS) in Cornwall, London, and Sussex in the UK [8]. They compared helicopter-attended patients and ambulance patients. There were no improvements in response times and the time on scene was longer for helicopter attended patients. They concluded that survival of patients was not improved by HEAS, and it was costly and the health care benefits were minor. This may be due to shorter transport distances and high-quality roads making a fast intervention by ambulance possible.

Generally, distances in excess of 30 min land travel to definitive care are considered to support the use of air transport in preference of land ambulance [9-11]. According to this statement, the distances between municipalities in northern Norway indicate helicopter and airplane the preferable tool in many cases when the patient is severely critically ill or injured. On the other hand the more frequent use of RW should be further examined as we have no general indications (i.e. number of road accidents) of an increase in the number of injuries during the study period. In this setting the planned register of trauma patients will be beneficial. Looking at CVD, the recent improvements in outcome with regard to PCI in acute myocardial infarction may have influenced the increased use of RW. Looking at place of living, there has been a growing centralisation of the population during the last decades. This fact could in the future reduce the need for air ambulances as hospitals are located in the populated areas (main cities).

Helicopters have limitations. Rough weather condition with a risk of ice is a significant problem during wintertime. Haug and coworkers looked at the access of HEMS in the non-coastal areas of southern Helgeland (southern areas of northern Norway) and revealed an availability rate of only 40% in the time period between November and March [12]. This is in accordance with our findings of the lowest regularity (68%) in this region. Nielsen concluded based on this knowledge that the ambulance should always be kept in mind as a good alternative [13]. However, in the Norwegian Arctic, ambulances are no alternative as roads are almost nonexistent [14].

Quality of care is an important issue. Unfortunately, we have no database at the NNRHA that could elucidate this topic. A study by Caldow and colleagues reported the experience from the Scottish Airambulance Services for 2002 and 2003 employing a helicopter for transfers and primary scene responses in rural and island areas of Scotland [15]. They recommend the establishing of a formalised system to access suitably trained medical staff instead of an ad hoc situation. Furthermore, they stated that rapid sequence intubation skills were required for any medical staff undertaking retrieval work. This is in accordance with the manning of the air ambulances in northern Norway. Hopefully, future Norwegian quality of care registers (i.e. the national trauma register) may document improved quality of care.

Benchmarking between health care providers may be a way forward to achieve improved cost-effectiveness and quality of care. Krüger and colleagues [11] analysed the Scandinavian pre-hospital physician-manned Emergency Medical Services (EMS) and revealed that differences were mainly related to time variables, patient volume, and service area. The Danish and Swedish services had higher volumes of patient care encounters while the Finnish and Norwegian ones provided a wider variety of medical services. Looking at the number of missions performed by ambulance helicopters in our study (396 - 773 mission), this is in accordance with Swedish figures varying between 400 and 1,200 per helicopter [16]. The helicopter in the city of Gothenburg did about 1,000 missions a year, with a total annual time in the air of 660 h. The corresponding maximum total time airborne of the Tromsø located AH was 689 h.

Patient transport by air is of great importance in Iceland because of its many sparsely populated areas and long distances [17]. There are approximately 450 fixed-wing ambulance missions annually. They have one dedicated air ambulance airplane located at Akureyri. In addition there are planes stationed in Westman Islands and Ísafjörður. These planes are manned on an ad hoc basis. Furthermore the coast guard runs three rescue helicopters (Aerospatiale Dauphin and Aerospatiale Super Puma). Thus six air ambulance resources serve a population of 300,000 inhabitants. This is somewhat less than our 11 units serving a population of 460,000 inhabitants in our region, but the geographical area of northern Norway is greater than Iceland. In comparison Scotland has a population of 5 million. There are two helicopters (Eurocopter 135) and two fixed-wing pressurized aircraft (King Air) for the sole use of the Scottish Ambulance Service, which, when required, may be backed up with Ministry of Defense and Her Majesty's (HM) Coastguard helicopters and other shared aircraft [17].

Looking to Sweden, the four northern counties (Västernorrland, Jämtland, Västerbotten, and Norrbotten) of Sweden share two fixed-wing air ambulances, which are Beech King-Air 200 models [16,17]. The basic crew for each plane is similar to those in Norway. These aircrafts fly between 6,000-8,000 missions per year and the majority is planned secondary transports between hospitals. The population of the four northern Swedish counties in 2008 was in total 877,758 inhabitants and the figure can thus be calculated 8 missions/1,000 inhabitants. The Swedish figure is lower than ours and probably due to shorter distances, fewer people living in remote areas, better weather conditions, and excellent roads favouring ambulance cars. Furthermore, Norrbotten county has an ambulance helicopter based in Gällivare. This helicopter was employed in 375 missions (595 hours) in 2009. Of these operations, 57% were secondary transports. These figures are comparable to our RW ones.

Health care administrators should recommend benchmarking analysis to achieve maximum return of investments. In view of the increasing use of air transport, it is timely to consider means by which cost-efficiency can be improved. Activity data, as obtained and analysed in our study, can be implemented in future cost-effectiveness analysis. As an example a change of base of an aircraft may introduce significant savings and improved availability. The implementation of medical emergency motorcycle (MEM) assistance was analysed by Nakstand and colleagues [18]. They concluded a small but significant reduction in driving time when using an MEM instead of a car ambulance. Whereas the gain was of little clinical importance, MEM was cheaper to operate, but the cost-effectiveness was reduced as it could not operate 12 months a year and was driven in daylight only.

In conclusion, we have revealed an increased request for air transport (RW and FW operation). The trend of centralisation of specialised health care is likely to continue and consequently increase the need for air ambulance resources. A significant increase in the use of RW resources in our study should undergo further analysis with regard to costs and strategic considerations with regard to geographic location of air bases before further economic resources are allocated to this service. Finally, our data did not address the impact of additional flights on primary outcome. In the future, improved patient outcome could be an area of research, especially when the trauma register has been implemented.

## Competing interests

The authors declare that they have no competing interests.

## Authors' contributions

Both TME and JN took part in the design of the study. TME collected the data from the LABAS database and presented an overview of the material. JN carried out the statistical analysis, searched the PubMed database for relevant studies/reports, and wrote the article. All authors read and approved the final manuscript.

## Authors' information

The author is a medical oncologist, professor at the Faculty of Medicine at the University of Tromsø, and medical director at the Northern Norway Regional Health Authority.

## References

[B1] Norum J Endresen E Injuries and diseases among commercial fishermen in the Northeast Atlantic and Barents Sea. Data from Royal Norwegian coast Guard Int Arch Occup Environ Health 2003 76 3 241 5 12690499 10.1007/s00420-002-0399-0

[B2] Norwegian Medical Association Norwegian Index for Medical Emergency Assistance 2005 2.1 The Laerdal Foundation for Acute Medicine, Stavanger, Norway

[B3] Norum J Elsbak TM The ambulance services in northern Norway 2004-2008. Improved competence, more tasks, better logistics and increased costs Int J Emerg Med 2010 3 69 74 10.1007/s12245-010-0166-z 20606813 PMC2885266

[B4] Baxt WG Moody P The impact of rotorcraft aeromedical emergency care service on trauma mortality JAMA 1983 249 3047 51 10.1001/jama.249.22.3047 6854826

[B5] Mitchell AD Tallon JM Sealy B Air versus ground transport of major trauma patients to a tertiary trauma centre: a provincewide comparison using TRISS analysis Can J Surg 2007 50 129 133 17550717 PMC2384270

[B6] Ringburg AN Thomas SH Steyerberg EW Van Lieshout EM Patka P Schipper IB Lives saved by helicopter emergency medical services. An overview of literature Air Med J 2009 28 6 298 302 10.1016/j.amj.2009.03.007 19896582

[B7] McVey J Petrie DA Tallon JM Air versus ground transport of the major trauma patient: A natural experiment Prehosp Emerg Care 2010 14 45 50 10.3109/10903120903349788 19947867

[B8] Snooks HA Nicholl JP Brazier JE Lees-Mlanga S The costs and benefits of helicopter emergency ambulance services in England and Wales J Publ Health Med 1996 18 67 77 10.1093/oxfordjournals.pubmed.a024465 8785079

[B9] Wills VL Eno L Walker C Use of an ambulance-based helicopter retrieval service Aust N Z J Surg 2000 70 506 510 10.1046/j.1440-1622.2000.01893.x 10901579

[B10] Cameron PA Helicopter transport: can physicians save lives? Aust N Z J Surg 1999 69 690 691 10527341 10.1046/j.1440-1622.1999.01687.x

[B11] Krüger AJ Skogvoll E Castrèn M Kurola J Lossius HM Scandinavian pre-hospital physician-manned Emergency Medical Services--same concept across borders? Resuscitation 2010 81 4 427 33 10.1016/j.resuscitation.2009.12.019 20122784

[B12] Haug B Avall A Monsen SA Reliability of air ambulances. A survey in three municipalities in Helgeland J Nor Med Assoc 2009 129 11 1089 1093 10.4045/tidsskr.08.0306 19488089

[B13] Nielsen EW Keep the feet on the ground J Nor Med Assoc 2009 129 11 1088. 10.4045/tidsskr.09.0598 19488088

[B14] Norum J Elsbak TM Airambulance services in the Arctic 1999-2009. A Norwegian study Int J Emerg Med 2011 4 1 10.1186/1865-1380-4-1 21407997 PMC3051888

[B15] Caldow SJ Parke TRJ Graham CA Munro PT Aeromedical retrieval to a university hospital emergency department in Scotland Emerg Med J 2005 22 53 55 10.1136/emj.2004.016618 15611548 PMC1726520

[B16] Björnstig U Prehospital emergency care in Sweden - with special emphasis on care of traffic victims IATSS Res 2004 28 24 31

[B17] Gunnarsson B Svavarsdóttir H Dúason S Sim A Munro A McInnesc C MacDonald R Ängquist KA Policy and service delivery. Ambulance Transport and Services in the Rural Areas of Iceland, Scotland and Sweden J Emerg Primary Health Care 2007 5 1 12

[B18] Nakstad AR Bjelland B Sandberg M Medical emergency motorcycle - is it useful in a Scandinavian Emergency Medical Service? Scand J Trauma Res Emerg Med 2009 17 9 10.1186/1757-7241-17-9 PMC2652419 19239681

